# Integrated Sensory, Nutritional, and Consumer Analysis of Sunflower Seed Butter: A Comparative Study of Commercial and Prototype Samples

**DOI:** 10.3390/foods14101815

**Published:** 2025-05-20

**Authors:** Kristel Vene, Evelyn Lumi, Maria Alas, Lachinkhanim Huseynli

**Affiliations:** 1 Department of Chemistry and Biotechnology, Tallinn University of Technology, Akadeemia tee 15, 12618 Tallinn, Estonia; kristel.vene@taltech.ee (K.V.); evelyn@aio.bio (E.L.); 2 Center of Food and Fermentation Technologies, Maealuse 2/4, 12618 Tallinn, Estonia; mariaalaswork@gmail.com

**Keywords:** sunflower butter, consumer preference study, descriptive sensory analysis, sensory profiling

## Abstract

Sunflowers (*Helianthus annuus* L.), traditionally cultivated for their oil, are increasingly valued for their nutritional and functional properties across a range of food applications. Sunflower seed butter is a nutritious, allergen-free alternative to traditional nut butter. Nevertheless, comprehensive information on its sensory properties and consumer acceptance is limited. This study aimed to evaluate the sensory characteristics, nutritional composition, and consumer preferences of sunflower seed butter, including commercial products and laboratory-developed prototypes. A total of 13 samples (11 commercial, 2 prototypes) were evaluated for protein, fat content (Kjeldahl, Soxhlet methods), and texture attributes, including hardness, stickiness, and spreadability. Descriptive sensory analysis was conducted by a trained panel (n = 10), and consumer acceptance was evaluated by 98 participants using a 9-point hedonic scale. The results indicated that consumer liking was primarily driven by flavor, particularly a roasted flavor profile with brown color and creamy texture. No significant correlations were found between consumer liking and protein or fat content. These findings underscore the dominant role of sensory attributes in shaping consumer perception and provide a basis for optimizing product formulation and marketing strategies in sunflower seed butter development.

## 1. Introduction

The sunflower crop, scientifically known as *Helianthus annuus* L., is one of the oldest oil plant species from North America, with evidence of cultivation dating back to 3000 BC [1]. Nowadays, sunflowers are primarily grown for oil production, with global output reaching approximately 22.2 million metric tons in the 2023/2024 marketing year, making it one of the major vegetable oils worldwide [2]. In addition to oil production, sunflower seeds are used as food, animal feed, for ornamental purposes, pharmaceuticals, cosmetics, biofuel, etc. The widespread popularity and extensive usage of sunflower seeds are due to their rich array of nutrients, including protein, unsaturated fats, fiber, vitamins, selenium, copper, zinc, folate, and iron [3]. According to studies, sunflower seeds consist of an average of 33.85% proteins, 65.42% lipids, and 2.73% ash, with the majority of these components concentrated in the kernels [4].

Beyond their traditional uses, sunflower seeds have also gained attention for their use in plant-based butter and spreads. The growing consumer demand for plant-derived alternatives has led to the development of nut and seed-based butter, which are recognized for their high nutritional value, including dietary fiber, proteins, and essential fatty acids. These products align with the increasing shift toward vegetarian and vegan diets, offering functional and health-promoting benefits [5,6]. Among these butter alternatives, sunflower butter provides a creamy and nutritious spread made from roasted sunflower seeds and additional ingredients such as sugars, salts, and oils [7]. Sunflower butter’s popularity is also driven by a growing number of individuals with tree nut allergies seeking a delectable and safe alternative [8,9]. The nutritional attributes closely resemble peanut butter, featuring eight times more vitamin E and four times more iron [10]. The choice to compare sunflower butter to peanut butter is strategic, as peanut butter serves as a familiar benchmark for most consumers. This comparison helps to highlight the differences and potential advantages of sunflower butter, particularly for those with allergies to nuts. Therefore, premium sunflower seed butter shows great promise as a potential ingredient for cookies, candies (similar to Reese’s), dairy-free spreads, high-protein snack bars, salad dressings, pestos, etc. Rich in antioxidants such as tocopherols and phenolic compounds, sunflower seeds processed into butter may enhance oxidative stability and protein content in food products, supporting shelf life, health, and protein demand [11,12,13]. However, the potential of sunflower butter remains underexplored in recent sensory studies, offering a compelling opportunity for further research. The most recent analysis of sunflower butter’s sensory profile was completed in 2005 [14], with a previous examination conducted in 1983 [15] in the United States, and no comparable research has been performed within the European Union. In the study conducted by Dreher et al. [15], sunflower butter was found to receive lower ratings when compared to peanut butter. Considering the recent technological advancements that have led to the production of superior-quality sunflower products, previous preferences might be influenced.

Given the limited research on sunflower butter, our objective in this study was to explore the attributes and consumer preferences of sunflower seed butter as a potentially valued product. The research was conducted with the help of a trained panel to perform sensory descriptions and comparisons among commercially available and lab-developed sunflower butter. Furthermore, a consumer preference study was carried out. Additionally, certain physical-chemical analyses were conducted. Considering that protein and fat content influence texture and mouthfeel in butter-like products [16], these components were also examined to better understand their potential contribution to sensory perception. This research aims to fill the gap in existing knowledge and provide insight into the sensory attributes and consumer preferences of sunflower seed butter [17].

## 2. Materials and Methods

### 2.1. Sample Preparation

Eleven commercially available sunflower seed butters were purchased from Amazon and retail shops (Table 1). For recipe development, raw sunflower seeds were provided by Letofin AS, originating from Ukraine. Additives (Sodium ascorbate, emulsifiers from Kerry Group (Ireland) and Puratos Group (Belgium), unrefined cane sugar, and sea salt) were selected based on their properties to mimic the sweetness, saltiness, and smoothness of butter spreads.

Seven hundred grams of raw sunflower seeds were soaked in a 2% sodium ascorbate solution for 30 min, stirring and keeping the temperature at 75–80 °C. After rinsing with distilled water, the seeds were roasted at 180 °C for 40 min, stirring occasionally for the roasting to be even. The roasted seeds were weighed, 8% sugar, 0.8% salt, and 1.5% emulsifier Purato (for “N42”) or 2% emulsifier Admul (for “N44”) were added based on weight. The mixture was stirred for another half hour after the addition of the emulsifier.

### 2.2. Protein Content

For the determination of protein content, the Kjeldahl method was employed in this study, using Velp Scientifica UDK 127 (Usmate, Italy) [18]. Three replicates were made for each sample.

### 2.3. Fat Content

The determination of crude fat content was conducted utilizing the Soxhlet method, exemplifying a thorough methodology for precise fat quantification [19]. The extractor used was a Velp Scientifica SER 148 (Usmate, Italy), and the solvent for the extraction was hexane. For each sample, three replicates were made.

### 2.4. Texture Analysis

The TA-XT2i Stable Micro Systems texture profile analyzer was used for the measurement of hardness, stickiness, and spreadability. For texture analysis, an average of 3 replicates was obtained. The sunflower butter was placed in a plastic container (35 mm diameter, 40 mm depth) to the top. Cone probe P/45C traveled downward through the sample at 1 mm/s, penetrating to a 20 mm depth and returning upward at the same speed, to the initial position. Hardness was evaluated by determining the maximum force of penetration, expressed in Newtons (N), which corresponds to the height of the positive peak on the force-deformation curve. The results were analyzed using the Texture Expert Exceed program.

### 2.5. Sensory Analysis

Consumer Analysis. The panelists (n = 98) were from Tallinn (Estonia), aged between 15 and 70 (68 were women, 30 were men). The panel represented a diverse range of dietary habits, including vegan. Five participants reported lactose intolerance, and two had hazelnut allergies. Most participants had limited prior exposure to sunflower seed butter; however, many were familiar with other plant-based spreads. Their overall consumption of nut and seed butters varied: 19% regularly consumed nut and seed butters (e.g., peanut, cashew, almond butter, and tahini) at least once a week, 36% consumed them 1–3 times per month, 37% reported intake between 1–6 times per year, and 8% consumed them less than once annually. This range of experience provided a balanced perspective on the sensory attributes of the product. Ten grams of the sunflower butter samples were placed into plastic cups coded with 3-digit random numbers for sample evaluation to ensure anonymity during the assessment process. The samples were presented to the panelists in a randomized order to mitigate order bias. Samples were served with water and crackers on a plastic tray to maintain consistency in presentation. Multiple sensory evaluation sessions were conducted to ensure comprehensive sensory feedback. Panelists were asked to rinse their mouths with water between samples to prevent flavor carry-over and ensure an accurate assessment of each sample. Panelists were instructed not to compare and rate the samples but to express their liking or disliking of each sample independently. It was emphasized that two completely different samples could be equally liked. A 9-point hedonic scale ranging from 1 dislike extremely to 9 like extremely was used to evaluate preference for the attributes of appearance, color, odor (smell), flavor, texture, spreadability, stickiness, and overall liking.

Descriptive Analysis. The descriptive sensory analysis was carried out by the sensory panel of the Center of Food and Fermentation Technologies. The analysis took place in a controlled environment, free from any disruptive odors, adhering to the guidelines outlined in ISO standard 8589:2007. Ten assessors (10 females, aged between 22 and 51 years) with a background in evaluating plant-based products participated in the sensory evaluation. All panelists were trained, experienced, and undergo regular monitoring. Before qualifying, each panelist successfully participated in a taste sensitivity test and demonstrated the ability to identify common food flavors.

Odor and flavor attributes were selected based on [14,20]. Following comprehensive discussion and training sessions with the panelists, only pertinent attributes were selected for the evaluations, as listed in Table 2. Additionally, an optional comment box was provided, enabling assessors to offer detailed descriptions of odor and flavor characteristics.

The sensory analysis sessions were conducted on separate days, during which the samples were assessed in two parallel sessions. Each session comprised the evaluation of three to four samples, completed within a half-hour timeframe. To ensure the panelists’ palates remained neutral between samples, they were provided with spring water and crackers for cleansing purposes.

The samples were served in 10 g portions and were assigned unique codes, with randomized presentation order using Williams’ Latin Square design. A numerical scale ranging from 0 to 9 was employed, with “0” indicating undetectable, “1” as low, “5” as medium, and “9” indicating high intensity. Sensory data was collected using the RedJade software (https://redjade.net/) developed by RedJade Sensory Solutions LLC, based in Martinez, CA, USA.

### 2.6. Statistical Analysis

The correlation analysis of consumer liking and physical-chemical analysis was done using MS Excel (version 2503). The correlation coefficient ranges from −1 to +1. The significance of differences was determined using one-way analysis of variance (ANOVA). Analyses were performed in SPSS version 26 (*p* < 0.05).

## 3. Results and Discussion

The research results indicate significant (*p* < 0.05) variations in both protein and fat content among the sunflower seed butter products. These diverse nutritional profiles among brands are likely influenced by differences in sunflower raw material quality and processing conditions. For instance, sample “Monki creme de tournesol” contains 25.7 g protein, in contrast to 4.8 g protein in “Wild Friends organic honey sunflower butter”. Similarly, fat content was 58.9 g in the “Dattelmann sunflower seed butter organic” sample compared to 14.2 g in “Dastony Sprouted sunflower seed butter”. Table 3 illustrates the protein and fat content of various sunflower butter samples.

While the protein and fat content provide insight into the nutritional value, these components also influence the texture attributes of the sunflower butter. Texture analysis of sunflower butter samples on hardness, stickiness, and spreadability is shown in Table 4. One of the key features of nut and seed butter is its spreadability. It is essential that the product maintains a soft consistency to prevent ripping bread or shattering crackers [16]. Stabilizers or emulsifiers are incorporated during product development to preserve the desired qualities of sunflower butter, as they influence the structural properties of the butter. Stabilizers create a structure that prevents oil from separating, ensuring uniform butter consistency. Studies show that hydrogenated oils and wax-based stabilizers, such as hydrogenated cottonseed oil, enhance oil binding, improve structural integrity, and can effectively maintain peanut butter consistency over time [21,22]. However, at times, these stabilizers can cause a repelling effect, resulting in a harder texture and reduced spreadability [23]. Grind size is another crucial factor that affects the texture, and study shows that grind size directly correlates with textural properties, such as decreased grind size increases spreadability, hardness, and stickiness [24]. This aligns with findings in sesame butter, where finer grinding led to increased viscosity, firmness, and consistency, with the smallest particle sizes producing the most stable and texturally desirable product [25].

Low hardness values indicate increased fluidity or less toughness, leading to better spreadability of sunflower butter [10]. These lower values may signify an optimal use of stabilizers or a different ratio in the product formulation. However, the texture characteristics of the “Monki creme de tournesol” sunflower sample stand out distinctly when compared to other samples. Its high hardness could be attributed to its high protein content. The samples’ spreadability might be influenced by a higher fat content, the particular processing techniques employed, as well as the specific emulsifiers or stabilizers used during the manufacturing process.

Sample “N42”, with its moderate hardness and spreadability, seems to align more closely with most of the commercial samples. However, “N44”, with its pronounced hardness and spreadability, was similar to the “SunButter creamy” sample. The distinct differences in texture profiles between “N42” and “N44” might be attributed to variations in the emulsifiers used in their formulations. Such differences further emphasize the diversity of formulation strategies among commercially available products.

**Figure 1:**
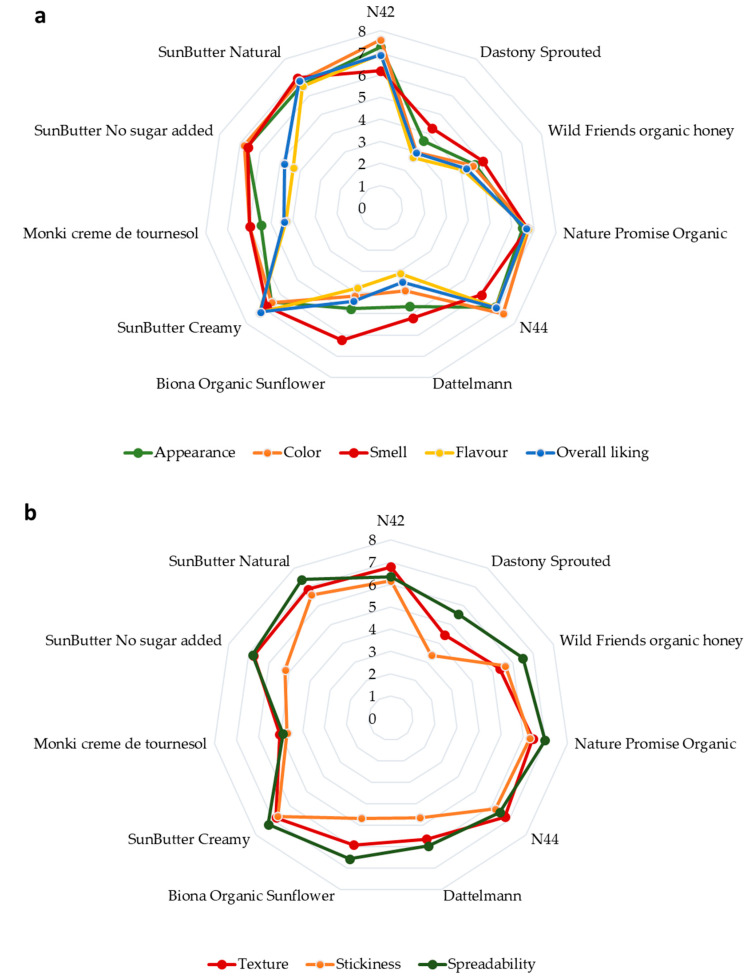
Spider web graph illustrating consumer evaluation of sunflower butter samples based on sensory attributes: (**a**) appearance, color, odor, flavor, and overall liking; (**b**) texture, spreadability, and stickiness (expressed 0–8 scale).

The results of a consumer study assessing various factors, such as appearance, color, texture, smell, flavor, stickiness, spreadability, and overall liking, are illustrated in Figure 1. Overall, consumer preferences varied across the samples, with attributes such as appearance, texture, and flavor playing significant roles in determining the liking of sunflower butter products. “SunButter Creamy” and sample “N42” were the top-rated sunflower butter options in the consumer study, followed by sample “N44” and “SunButter Natural” according to their overall liking. While “Dastony” and “Dattelmann” received the lowest ratings, suggesting that they were less preferred by the participants. Those sunflower samples received an especially low ranking on color and flavor attributes. Those variations in color and flavor can be due to the qualities of raw materials as well as the roasting [26]. Sunflower butter is generally accepted within a darker color range compared to most commercially available peanut butter [27].

The correlation between the extent of overall liking and the protein, lipid content, and texture attributes of the product is presented in Table 5. Texture parameters showed a strong correlation (1.0), meaning that harder textures were also stickier and less spreadable. However, there was no direct correlation between texture attributes and consumer liking. Spreadability (consumer) showed a negative correlation with hardness (−0.7) and stickiness (−0.6). Appearance (consumer) attribute showed a lack of correlation with protein and fat content but demonstrated stronger positive correlations with color, texture, smell, and flavor, suggesting that visually appealing samples were generally rated more positively across other sensory domains. Protein and fat content varied a lot between samples, and there was minimal to no correlation between overall liking. The lack of correlation between liking and nutritional content suggests that while consumers may value nutritional aspects, these are not the primary drivers of sensory acceptance [28]. This aligns with the well-established understanding that sensory attributes dominate consumer purchase decisions and satisfaction, often outweighing nutritional considerations [29,30].

The possible explanation for this lack of correlation may stem from protein-flavor and lipid-flavor interactions. The protein content can enhance flavor binding, potentially delaying volatile release and modifying flavor perception, while lipids may modulate flavor volatility, influencing the overall aroma and taste profile [31]. This suggests that, rather than a true lack of correlation, the absence of a direct link between overall liking and protein/lipid content may result from complex interactions involving flavor, texture, and sensory perception, warranting further investigation.

The high correlation between flavor and overall liking (correlation 1.0) following appearance and color (correlations both 0.9) underscores the important role of flavor in consumer satisfaction. This aligns with previous studies on walnut butter, where appearance, flavor, and consistency played a significant role in product acceptance [32].

A descriptive sensory analysis was conducted using a trained panel to understand further the sensory characteristics driving consumer preferences. This method provides valuable insights into the key sensory attributes [33], enabling the optimization of the product to meet the needs and expectations of the consumers [34]. Flavor, appearance, odor, and texture attributes showed variation across the samples, according to panel scores. However, some of the samples showed similarity in their attributes and aligned closely. Such as “N42” and “N44” shared common attributes, including denser texture, higher cohesiveness, and a notable presence of sweetness, saltiness, and strong overall flavor intensity.

**Figure 2:**
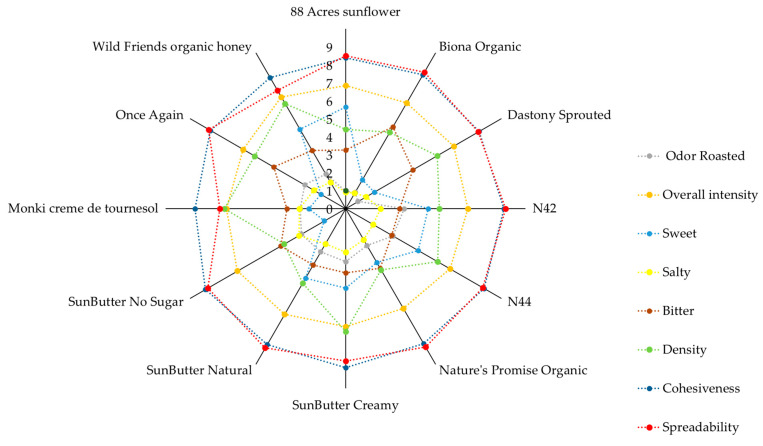
Descriptive sensory analysis (0–9 scale) of sunflower butter samples, visualized as a spider web graph. Part of the full dataset is presented to ensure clarity; complete data can be found in the Supplementary Material.

Moving on to “SunButter Creamy” and “SunButter Natural”, both samples exhibit dense and cohesive textures with minimal graininess. “SunButter Creamy” leans toward a less sweet flavor profile compared to “SunButter Natural”. Among the samples, “Wild Friends Organic Honey” was distinctly different with its unique flavor profile, characterized by a pronounced sweetness and a prominent honey flavor. “Nature’s Promise Organic” was marked by “oiliness” and “lower density” compared to other samples, setting it apart in terms of texture. “Biona Organic Sunflower Seed Butter”, on the other hand, differs from the lab samples in terms of sweetness, saltiness, and texture attributes (Figure 2).

This sample also featured flavor descriptors such as “chemical”, “green”, and “oily”, which may indicate the presence of unusual or off-putting flavor notes, potentially resulting from lipid oxidation. Similar off-flavors, particularly “grassy” and “beany” notes, have also been reported in soy butter products, which were associated with lipoxygenase activity and oxidation of unsaturated fatty acids [5,35]. In the case of peanut butter, the study observed that prolonged storage leads to the development of additional off-flavors, including “cardboard”, “painty”, and “rancid” [36].

“Dastony Sprouted” emerges as a unique sample with a characteristic unpleasant taste. It distinguishes itself with a crunchier texture and an unpalatable flavor described as “fungus” and “halva”. The sample differed by the fact that the seeds were sprouted before the spread production. In soy butter, sprouting before roasting has been shown to improve flavor by reducing off-notes [35]. However, in this sample, the development of undesirable flavors may be the result of other processing factors or raw materials.

Overall, the evaluations suggest that butter made from roasted sunflower seeds was preferred, which was characterized by a roasty aroma, brown color, balanced taste, and creamy texture. These samples exhibited overall high aroma intensity, along with the characteristic sunflower seed flavor. “SunButter Creamy”, “SunButter Natural”, “N42”, and “N44” are good examples of this preference. Similarly, in the cashew nut butter study, consumers preferred samples with a roasted aroma and smooth texture [37]. In contrast, products that were characterized by strong bitter, green, astringent, and rancid flavors were generally disliked. According to studies, to improve these kinds of butter off-flavors, methods such as optimized roasting, sprouting, selecting suitable kernel varieties, and maintaining proper storage conditions have been widely used [25,35,36]. These findings emphasize that the role of processing methods plays a crucial role in shaping the sensory properties of sunflower butter, influencing both flavor development and overall consumer acceptance.

With growing health awareness, the demand for lower-fat, lower-sugar nut and seed butter products is expected to increase as consumers seek healthier alternatives without compromising sensory appeal. In this context, studies on pistachio butter have explored the use of fat and sweet replacers to develop healthier, lower-calorie formulations [38,39]; however, similar research has not yet been conducted for sunflower butter. This gap presents an opportunity to leverage sensory research in optimizing formulations that achieve nutritional improvements while delivering a positive sensory experience.

## 4. Conclusions

This study highlights the diverse sensory profiles and consumer preferences associated with sunflower butter products. The findings demonstrate that while texture parameters such as hardness, stickiness, and spreadability are crucial, they do not directly correlate with overall consumer liking. Instead, flavor, appearance, and color emerged as the primary drivers of preference. The variation in protein and lipid content among samples suggests the influence of different processing techniques and raw material quality. This research provides valuable insights for manufacturers aiming to align their products with consumer expectations, emphasizing the need for continued innovation and adaptation to evolving market trends. Future studies should focus on exploring the impact of specific ingredients and processing methods on sensory properties and consumer acceptance of sunflower butter.

## Figures and Tables

**Table 1:** Ingredients of sunflower butter samples, including commercial and lab-formulated (N42 and N44) sunflower butter samples.

Sunflower Butter Sample	Ingredients
SunButter creamy (Sun Butter LLC, Fargo, ND, USA)	Roasted sunflower seeds, mono- and di-glycerides, sugar, and salt.
Nature’s Promise Organic Sunflower (Foodhold USA, LLC, Landover, MD, USA)	Dried organically grown sunflower seeds, organic sugar, organic sunflower oil, and salt.
SunButter, no sugar added (SunButter LLC, Fargo, ND, USA)	Roasted sunflower seeds and salt.
Wild Friends organic honey sunflower butter (Wild Friends, Portland, OR, USA)	Organic roasted sunflower seeds, organic sunflower oil, organic clover honey, and sea salt.
Monki creme de tournesol (Monki, Horizon Natuurvoeding, IJsselstein, The Netherlands)	Roasted sunflower seeds from controlled organic farming and sea salt.
SunButter Natural (SunButter LLC, Fargo, ND, USA)	Roasted sunflower seeds, sugar, and salt.
Once again, sunflower seed butter (Once Again Nut Butter, Nunda, NY, USA)	Organic sunflower seeds and organic sunflower oil.
88 Acres sunflower (88 Acres, Canton, MA, USA)	Organic sunflower seeds, organic maple sugar, organic pressed sunflower oil, and sea salt.
Dattelmann sunflower seed butter organic (Dattelmann, Berlin, Germany)	100% roasted sunflower seeds from certified organic farming.
Biona Organic Sunflower Smooth (Biona, London, UK)	Freshly roasted seeds.
Dastony Sprouted sunflower seed butter (Windy City Organic, LLC, Northbrook, IL, USA)	100% certified organic sprouted sunflower seeds.
N42 (Letofin AS, Tallinn, Estonia)	Roasted sunflower seeds, emulsifier, sugar, and salt.
N44 (Letofin AS, Tallinn, Estonia)	Roasted sunflower seeds, emulsifier, sugar, and salt.

All samples were purchased in 2022.

**Table 2:** The selected attributes for descriptive analysis are used in this study.

Sensory Modalities	Descriptors	Definition	Reference Values
Appearance	Brown color	Intensity of brown hue in the product.	Light (0), Dark (9)
	Additional comments	Any additional visual characteristics or observations.	
Odor	Overall intensity	Strength or potency of the aroma.	Weak (0), Strong (9)
	Raw material	Natural scent of the plant-based raw materials.	Weak (0), Strong (9)
	Roasted	Aroma resulting from the roasting process.	Weak (0), Strong (9)
	Rancid	Perception of a stale or off-odor.	Weak (0), Strong (9)
	Additional comments	Any additional olfactory notes or observations.	
Flavor	Overall intensity	Strength or intensity of the overall flavor.	Weak (0), Strong (9)
	Raw material (seeds)	Natural flavor of plant-based seeds.	Weak (0), Strong (9)
	Roasted	Flavor profile resulting from the roasting process.	Weak (0), Strong (9)
	Sweet	Perception of sweetness.	Weak (0), Strong (9)
	Salty	Perception of saltiness.	Weak (0), Strong (9)
	Sour	Perception of acidity.	Weak (0), Strong (9)
	Bitter	Perception of bitterness.	Weak (0), Strong (9)
	Astringent	Perception of a puckering or drying sensation.	Weak (0), Strong (9)
	Rancid	Perception of a stale or off-flavor.	Weak (0), Strong (9)
	Additional comments	Any additional taste-related observations.	
Texture	Graininess	Presence of coarse particles or granules.	Weak (0), Strong (9)
	Hardness	Firmness or resistance to pressure.	Weak (0), Strong (9)
	Density	Compactness or thickness of the product.	Weak (0), Strong (9)
	Adhesiveness	The degree to which the product sticks to the surface.	Weak (0), Strong (9)
	Cohesiveness	The degree to which the product holds together.	Weak (0), Strong (9)
	Oiliness	Perception of oil or grease in the mouth.	Weak (0), Strong (9)
	Smooth	Absence of roughness or irregularities.	Weak (0), Strong (9)
	Ease of swallow	The effort required to swallow the product.	Easy (0), Hard (9)
	Mouthcoating	The degree to which the product coats the mouth.	Weak (0), Strong (9)
	Spreadability	Ease with which the product spreads.	Weak (0), Strong (9)
	Additional comments	Any additional textural observations.	

**Table 3:** Protein and fat content of sunflower butter samples analyzed in the laboratory. Different letters in columns indicate statistically significant differences (*p* < 0.05).

Sunflower Butter Samples	Protein Content, g/100 g	Fat Content g/100 g
SunButter creamy	8.9 ± 0.14 ^a^	29.5 ± 1.70 ^l,m^
Nature’s Promise Organic Sunflower	6.2 ± 0.21 ^b^	33.2 ± 0.78 ^m^
SunButter, no sugar added	11.4 ± 0.2 ^c^	49.6 ± 2.26 ^n^
Wild Friends organic honey sunflower butter	4.8 ± 0.14 ^d^	31.7 ± 1.27 ^l,m^
Monki creme de tournesol	25.7 ± 0.42 ^e^	53.6 ± 0.64 ^o^
SunButter natural	13.0 ± 0.14 ^f^	18.5 ± 0.42 ^p^
Once again, sunflower seed butter	11.4 ± 0.49 ^c^	21.6 ± 0.57 ^q^
88 Acres sunflower	12.0 ± 0.78 ^c^	17.2 ± 1.13 ^p^
Dattelmann sunflower seed butter organic	19.6 ± 0.35 ^h^	58.9 ± 0.07 ^r^
Biona Organic smooth	18.5 ± 0.28 ^i^	45.4 ± 0.64 ^s^
Dastony Sprouted sunflower seed butter	6.80 ± 0.28 ^b^	14.2 ± 0.57 ^p^
N42	19.8 ± 0.28 ^h,k^	28.8 ± 1.70 ^l^
N44	20.5± 0.28 ^k^	31.3 ± 1.56 ^l,m^

**Table 4:** Texture analysis of sunflower butter samples. Different letters in columns indicate statistically significant differences (*p* < 0.05).

Sunflower Butter Samples	Hardness, N	Stickiness, N	Spreadability, N·S
SunButter creamy	1.35 ± 0.10 ^b^	0.35 ± 0.02 ^d^	7.01 ± 0.99 ^g^
Nature’s Promise Organic Sunflower	0.26 ± 0.12 ^a^	0.09 ± 0.03 ^e^	1.06 ± 0.38 ^h^
SunButter, no sugar added	0.19 ± 0.04 ^a^	0.09 ± 0.02 ^e^	0.88 ± 0.17 ^h^
Wild Friends organic honey sunflower butter	0.07 ± 0.02 ^a^	0.02 ± 0.01 ^e^	0.37 ± 0.09 ^h^
Monki creme de tournesol	20,73 ± 1.60 ^c^	1,69± 0.26 ^f^	120.65 ± 9.86 ^i^
SunButter natural	0.12 ± 0.02 ^a^	0.04 ± 0.01 ^e^	0.56 ± 0.07 ^h^
Once again, sunflower seed butter	0.34 ± 0.06 ^a^	0.15 ± 0.03 ^e^	1.65 ± 0.21 ^h^
88 Acres sunflower	0.18 ± 0.03 ^a^	0.05 ± 0.02 ^e^	0.71 ± 0.11 ^h^
Dattelmann sunflower seed butter organic	0.19 ± 0.01 ^a^	0.09 ± 0.01 ^e^	0.84 ± 0.01 ^h^
Biona Organic smooth	0.14 ± 0.01 ^a^	0.05 ± 0.01 ^e^	0.60 ± 0.01 ^h^
Dastony Sprouted sunflower seed butter	0.08 ± 0.01 ^a^	0.02 ± 0.00 ^e^	0.34 ± 0.02 ^h^
N42	0.22 ± 0.06 ^a^	0.08 ± 0.03 ^e^	0.96 ± 0.22 ^h^
N44	1.50 ± 0.17 ^b^	0.33 ± 0.04 ^d^	6.99 ± 1.41 ^g^

The categorization of high, medium, or low values is based on relative comparisons among the tested samples.

**Table 5:** Correlation analysis of consumer liking in relation to composition and texture characteristics of the product.

	Hardness	Stickiness	Spreadability	Appearance (Consumer)	Color (Consumer)	Texture (Consumer)	Smell (Consumer)	Flavor (Consumer)	Stickiness (Consumer)	Spreadability (Consumer)	Overall Liking	Fat	Protein
Hardness	1.0												
Stickiness	1.0	1.0											
Spreadability	1.0	1.0	1.0										
Appearance (consumer)	−0.2	−0.1	−0.2	1.0									
Color (consumer)	0.1	0.2	0.1	0.9	1.0								
Texture (consumer)	−0.3	−0.2	−0.3	0.9	0.8	1.0							
Smell (consumer)	0.0	0.1	0.0	0.8	0.8	0.8	1.0						
Flavor (consumer)	−0.2	−0.1	−0.2	0.8	0.8	0.7	0.7	1.0					
Stickiness (consumer)	−0.1	−0.1	−0.1	0.7	0.7	0.7	0.7	0.8	1.0				
Spreadability (consumer)	−0.7	−0.6	−0.7	0.4	0.2	0.5	0.6	0.4	0.4	1.0			
Overall liking	−0.2	−0.1	−0.2	0.9	0.9	0.8	0.7	1.0	0.8	0.4	1.0		
Fat	0.4	0.4	0.4	−0.1	−0.1	0.1	0.1	−0.5	−0.1	−0.2	−0.3	1.0	
Protein	0.6	0.6	0.6	0.1	0.1	0.2	0.0	0.0	0.0	−0.5	0.0	0.5	1.0

## Data Availability

The original contributions presented in this study are included in the article/Supplementary Materials. Further inquiries can be directed to the corresponding author.

## Supplementary Materials

The following supporting information can be downloaded at: https://www.mdpi.com/article/10.3390/foods14101815/s1.
