# Supplementary figures and images for: Integrated Sensory, Nutritional, and Consumer Analysis of Sunflower Seed Butter: A Comparative Study of Commercial and Prototype Samples
Source: Foods. 2025 May 20;14(10):1815. doi: 10.3390/foods14101815 (PMC12111572; doi:10.3390/foods14101815)

## Appearance and Odor

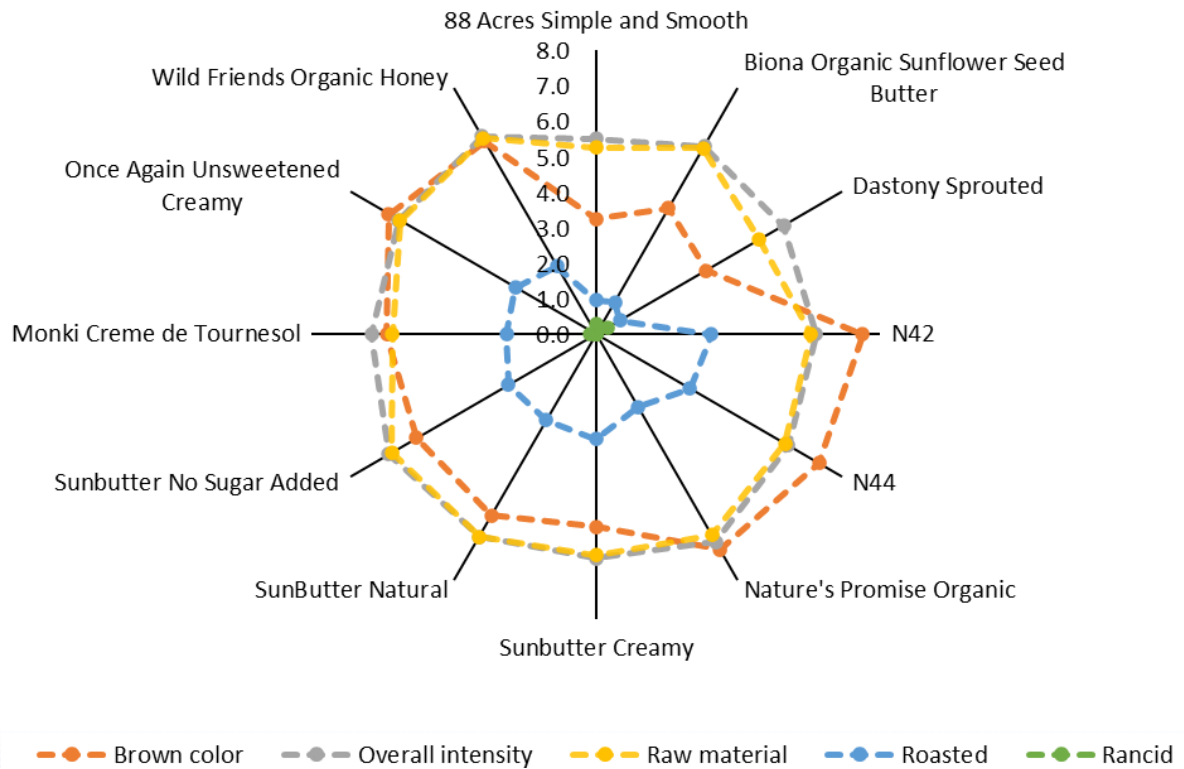

## Flavor

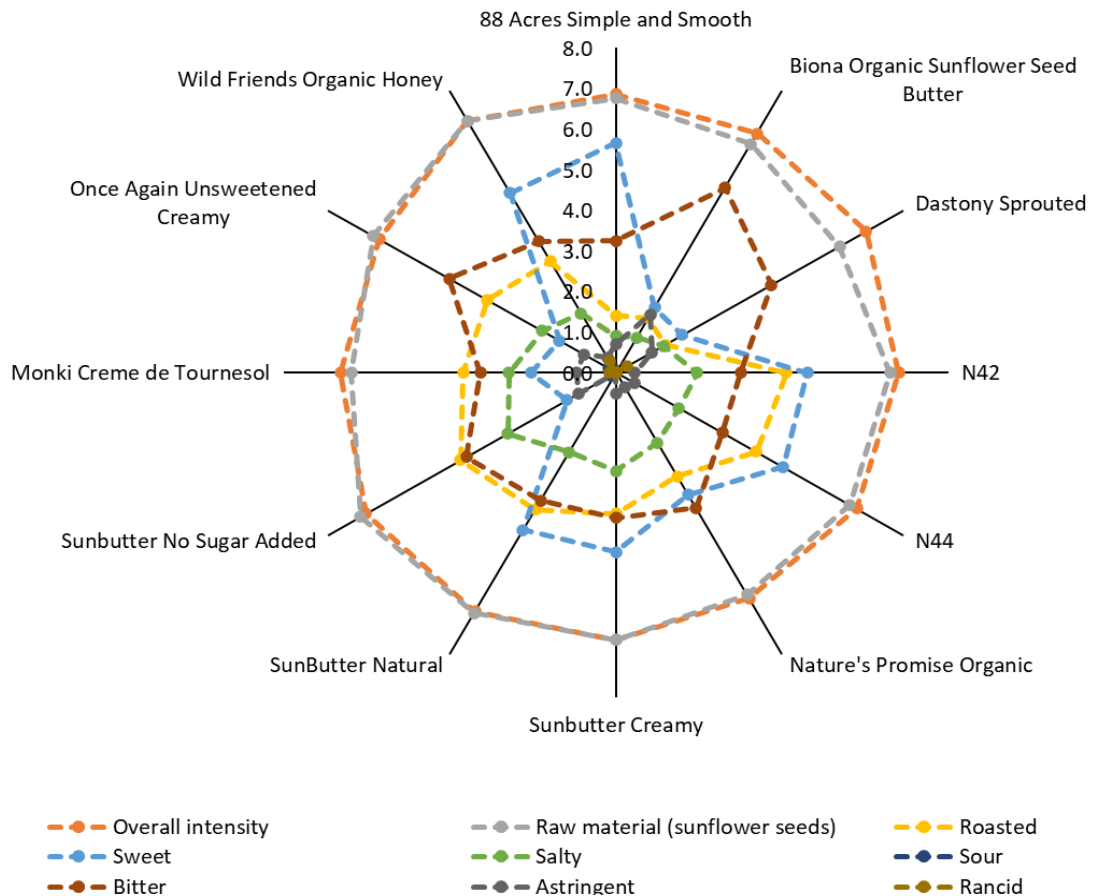

## Texture part 1

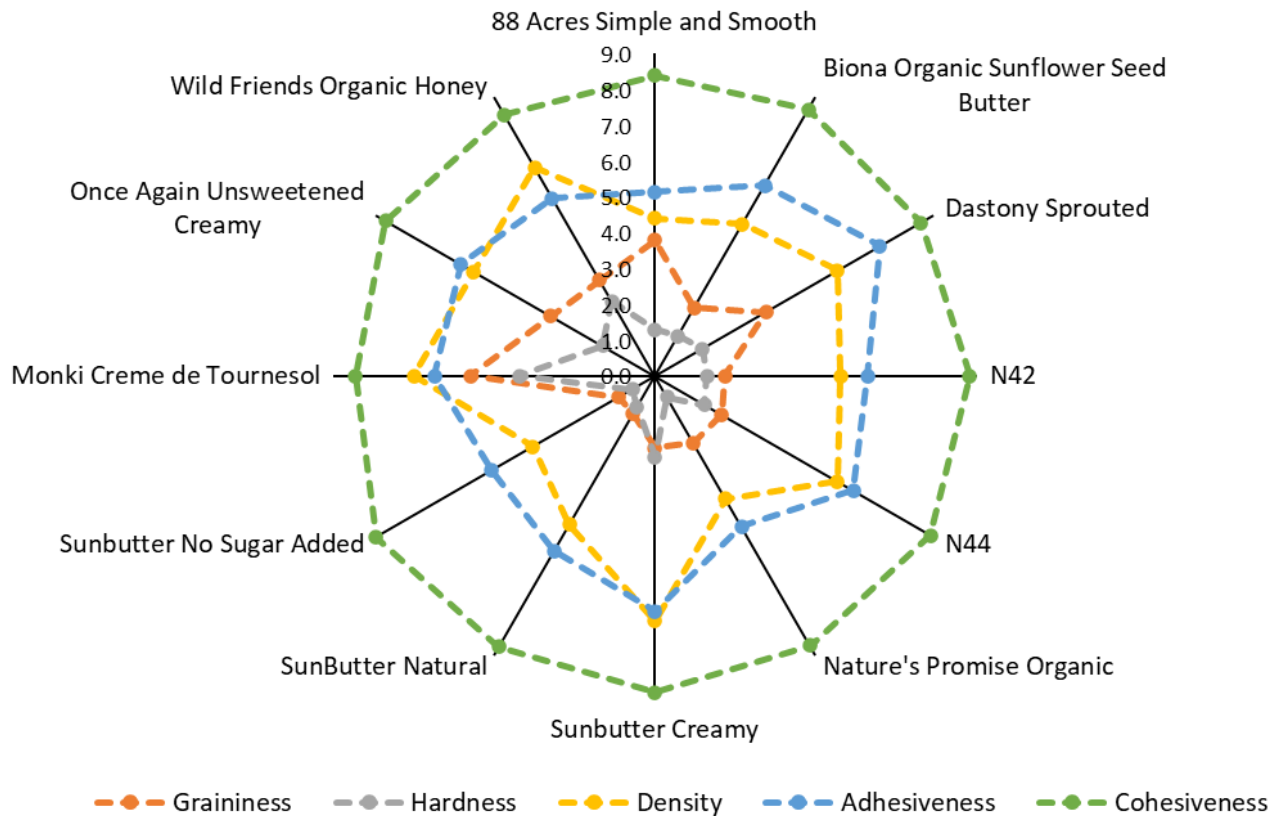

## Texture part 2

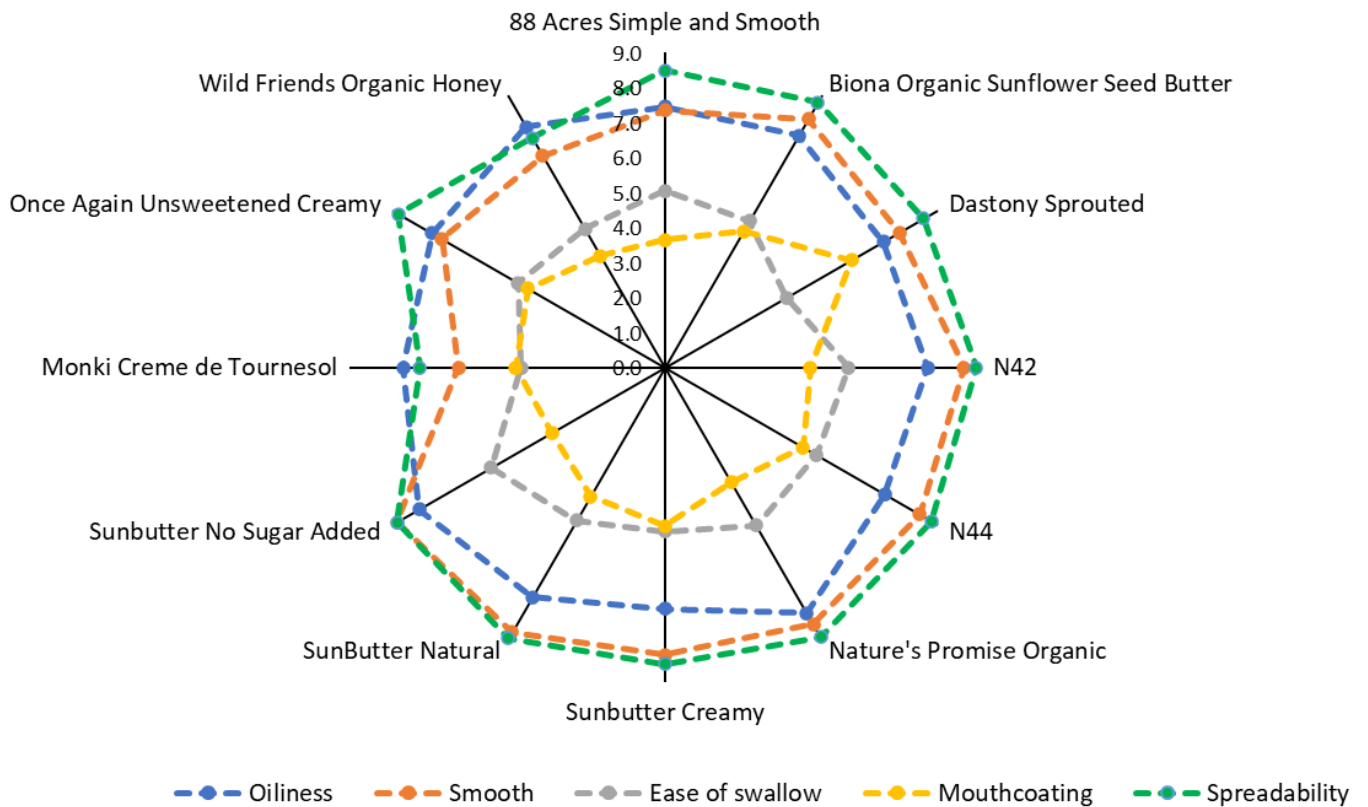

Supplement: Supplementary file 1 [file foods-14-01815-s001.zip › foods-3583142-supplementary.pdf]
